# Comparison of growth performance and tissue cobalt concentrations in beef cattle fed inorganic and organic cobalt sources

**DOI:** 10.1093/tas/txad120

**Published:** 2023-10-18

**Authors:** Rachel Raths, Brandon Rodriguez, Joseph W Holloway, Audie Waite, Ty Lawrence, Jennifer L G van de Ligt, Heb Purvis, Heidi Doering-Resch, David P Casper

**Affiliations:** Ralco Animal Nutrition, Marshall, MN 50258, USA; Ralco Animal Nutrition, Marshall, MN 50258, USA; Ralco Animal Nutrition, Marshall, MN 50258, USA; Agri-Research Center, Canyon, TX 79015, USA; Beef Carcass Research Center, Department of Agricultural Sciences, West Texas A&M University, Canyon, TX 79016, USA; ToxStrategies LLC, Katy, TX 77494, USA; Ralco Animal Nutrition, Marshall, MN 50258, USA; Ralco Animal Nutrition, Marshall, MN 50258, USA; Ralco Animal Nutrition, Marshall, MN 50258, USA

**Keywords:** acetate, carbonate, cobalt, growth, lactate, tissue residue

## Abstract

Cobalt is an essential trace mineral required for ruminal vitamin B_12_ synthesis, but sources differ in ruminal microbial utilization, i.e., cobalt carbonate is poorly water soluble, whereas acetate and lactate forms are water soluble. Reports comparing organic cobalt lactate to other cobalt salts are lacking. The study objective was to determine if feeding cobalt lactate at two inclusion rates resulted in similar growth performance and tissue cobalt concentrations as the carbonate and acetate forms used in feeds. One hundred Angus cross bred steers weighing 385 ± 20 kg were randomly assigned to one of five treatments. Cattle were fed a basal diet plus: 1) cobalt carbonate to supply cobalt at 30 mg/steer/d, 2) cobalt acetate to supply cobalt at 30 mg/steer/d, 3) cobalt acetate to supply cobalt at 60 mg/steer/d, 4) cobalt lactate to supply cobalt at 30 mg/steer/d, and 5) cobalt lactate to supply cobalt at 60 mg/steer/d. Cattle were fed according to industry standards until body fat deposition was visually deemed to grade USDA Choice, which was 92 and 117 d for each of the 2 blocks, respectively. Steers were harvested and carcass measurements recorded along with sampling of adipose, heart, kidney, liver, and muscle for tissue cobalt concentrations. Three statistical contrasts consisted of: 1: inorganic (cobalt carbonate) vs. organic (cobalt acetate and lactate); 2: cobalt acetate vs. cobalt lactate; and 3: feeding rate of 30 vs. 60 mg/steer/d cobalt. Body weight gains, average daily gains, dry matter intake, and feed conversions were similar (*P* > 0.10) for steers fed all cobalt sources and feeding rates. Hot carcass weight, yield grade, back fat thickness, and ribeye area were similar (*P* > 0.10) among steers fed all cobalt sources and inclusion rates. Liver, kidney, muscle, and adipose cobalt concentrations were similar (*P* > 0.08) for steers fed inorganic vs. organic cobalt sources. Feeding cobalt lactate compared with cobalt acetate did not affect (*P* > 0.10) liver, kidney, heart, muscle, and adipose tissue cobalt concentrations. Feeding 60 mg/steer/d cobalt compared with 30 mg/steer/d increased (*P* < 0.01) liver, kidney, heart, and adipose tissue cobalt concentrations, while muscle was a tendency (*P* < 0.06). The study demonstrated that feeding soluble cobalt lactate, a new cobalt source, resulted in similar growth performance, carcass characteristics, and tissue cobalt concentrations when compared with cobalt acetate and carbonate.

## Introduction

Cobalt is an essential trace mineral for ruminants (i.e., beef and dairy cattle) that is utilized by ruminal microbes for cobalamin (vitamin B_12_) synthesis (NRC, 2001, 2005; NASEM, 2021). Cobalt is recognized as a low concern for animal health and the maximum tolerable concentration was set at 25 mg/kg feed for ruminant species (NRC, 2005). Vitamin B_12_ is synthesized by ruminal microbes and is integral to several biochemical reactions. The most well-known cobalt metabolic reactions are blood glucose synthesis from ruminal propionate production, methionine synthesis, and DNA methylation (McDowell, 2017). When adequate dietary cobalt is available, typically through supplementation because most feed ingredients are low in cobalt (<0.5μg/kg; NRC, 2005), ruminal microbes can synthesize adequate vitamin B_12_ to meet the nutrient requirements for both the ruminal bacteria and the host animal (NASEM, 2016; Watamabe and Bito, 2018). However, increased dietary cobalt concentrations through supplementation has been reported to positively influence ruminal vitamin B_12_ synthesis and animal performance (Mills, 1981; Kawashima et al., 1997b; Akins et al., 2013).

For ruminants, ~3% of dietary cobalt is utilized for vitamin B_12_ synthesis, although up to 13% of dietary cobalt can be utilized when feeding insufficient cobalt (Smith and Marston, 1970). Walker and Elliot (1972) reported that compared to a high forage diet, a high grain diet altered ruminal fermentation toward reduced ruminal B_12_ synthesis but a greater synthesis of vitamin B_12_ analogs, which are not biologically active. Sutton and Elliot (1972) also reported that feeding a high forage diet tended to promote greater vitamin B_12_ production, increasing the vitamin B_12_ ratio to other B_12_ analogs.

Previously, the dietary guideline for meeting the ruminant cobalt nutrient requirement was 0.10 mg/kg dry matter for beef (NRC, 2000) and 0.11 mg/kg dry matter for dairy cattle (NRC, 2001), which is based on supplying sufficient dietary cobalt for maintaining tissue B_12_ concentrations above 0.3 µg/L (NRC, 2001). Mills (1981) reported that vitamin B_12_ synthesis increased 20-fold in sheep when dietary cobalt concentrations increased from 0.1 to 0.5 mg/kg dry matter. Tiffany et al. (2006) reported increased vitamin B_12_ synthesis when dietary cobalt concentrations increased from 0.1 to 1.0 mg/kg using an in vitro fermentation system. Recent cattle dietary cobalt guidelines were increased to 0.15 mg/kg for beef cattle (NASEM, 2016) and 0.20 mg/kg for dairy cattle (NASEM, 2021), but these guidelines were based on a constant cobalt bioavailability and guidelines do not vary by solubility of the cobalt source. This variation seems to provide a large range of recommended cobalt feeding concentrations for ruminants regardless of what the current NRC (NASEM, 2016, 2021) indicates as recommended requirements. In 2015, a survey of the nutritional recommendations of feedlot consulting nutritionists found that the majority of these respondents, consulting for over 14 million beef cattle, provided trace minerals at numerical concentrations at or above NRC (2000) recommendations (Samuelson et al., 2016). Of these same respondents, over 77.3% recommended using a combination of both organic and inorganic trace mineral forms in receiving rations and over 54.6% in finishing rations. Cobalt trace mineral concentrations were reported to average 1.03 mg/kg with a maximum inclusion of 5.0 mg/kg in receiving rations and average 0.82 mg/kg with a maximum inclusion of 3.0 mg/kg in finishing rations. This report had similar results to a survey conducted in 2007 in which trace minerals were typically fed within one to two times the NRC (2000) recommendations for beef cattle (Vasconcelos and Galyean, 2007).

Evidence exists that dietary cobalt form impacts the efficiency of ruminal microbial vitamin B_12_ synthesis and fiber digestion and other ration components, mainly the forage to concentrate ratio, may or may not impact tissue cobalt concentrations (Tiffany et al., 2003; González-Montaña et al., 2020). Studies looking at both water solubility of the differing cobalt forms and concentrations supplemented above (NRC, 2005) maximum tolerable concentrations of cobalt are not well researched. Cobalt acetate, carbonate, chloride, nitrate, propionate, sulfate, and glucoheptonate all appear to be adequate cobalt sources for ruminants (McDowell, 2017) to meet the cobalt nutrient requirement, but water solubility varies tremendously among sources (Tiffany et al., 2003). Cobalt water solubility affects cobalt availability for ruminal digestion and microbial B_12_ synthesis with organic forms generally having greater ruminal solubilities resulting in more bioavailability than non-organic forms (Gayathri and Panda, 2018). For example, cobalt oxide is less soluble resulting in lower conversions to vitamin B_12_ during ruminal in vitro fermentations (Kawashima et al., 1997b). Kawashima et. al. (1997b) reported that the water solubility of cobalt sources used in his experiment was 100, 72, 1.5 and less than 1 for the following sources: sulfate, glucoheptonate, carbonate, and oxide, respectively. Kawashima et al. (1997a) reported that cobalt oxide forms were less available than other cobalt forms when supplemented in sheep. Cobalt acetate was reported to be similar in bioavailability to cobalt carbonate, cobalt glucoheptonate (Tiffany et al., 2003) and cobalt sulfate (Nabhushaka et al., 2005).

However, outside of these examples, there is a paucity of published research evaluating different cobalt sources, their solubility, and bioavailability. The available cobalt sources approved as feed supplements in the United States include acetate, carbonate, and sulfate forms, but the sulfate form is usually not commercially economical. Cobalt carbonate is an inorganic source of cobalt while cobalt acetate and lactate are organic sources of cobalt. Cobalt carbonate, the most widely used form for cattle supplementation in the United States, has low water solubility, around 1.5 relative solubility according to Kawashima et al. (1997b). Cobalt acetate is very soluble but is not readily available in the marketplace. Cobalt lactate is a new source of cobalt that is water soluble (4.5 g/100 mL) and highly bioavailable, but is not currently an approved feed supplement in the United States.

This study was conducted to directly compare supplementation with cobalt lactate to supplementation with cobalt carbonate or cobalt acetate and hypothesized that cobalt source would have minimal impact on growth performance, tissue, and serum cobalt concentrations. Cobalt supplemented amounts at 30 mg/steer/d are higher than those concentrations recommended by the NRC (NASEM, 2016) for beef cattle but within the range that Samuelson et al, (2016) reports in both receiving and finishing feedlot rations. The 60 mg/steer/d supplement amount is twice that of the suggested cobalt supplemented amount required in this study by the Food and Drug Administration Center for Veterinary Medicine. A concentration higher than NRC, 2016 recommended, but much lower than the maximum tolerable concentration of cobalt, was supplemented based on the study goals in addressing not only growth performance but also serum and tissue concentrations. The findings that feedlot rations may not meet the cobalt requirements of finishing cattle when fed higher corn sources, i.e., higher starch, lower fiber rations, provided the background for the 30 mg/steer/d base amount (Stangl et al., 2000; Tiffany et al., 2003). Tiffany et al. (2003) indicate that in receiving rations, corn is the predominant grain used in both receiving and finishing rations (87.5% and 100%, respectively). Processing methods for feedlot rations also include highly fermentable grains with 65.2% and 70.8% of receiving and finishing rations using steam flaked corn as their primary processing method followed by dry rolled corn at 43.5% and 43.5%, respectively. This study evaluated the effect of feeding cobalt carbonate at 30 mg/steer/d, vs. feeding the cobalt salts, cobalt acetate, and cobalt lactate, at two inclusion rates (30 mg/steer/d and 60 mg/steer/d), on growth performance, carcass characteristics, serum, and tissue cobalt concentrations in finishing beef cattle.

## Materials and Methods

### Animal care and management

The growth performance feedlot trial was conducted at Agri Research Center Inc. (Canyon, TX) from July 5 through December 13, 2021. All animals were cared for and managed according to the Beef Quality Assurance guidelines as certified by the Texas Cattle Feeders Association through biennial onsite inspections (Texas Cattle Feeders Association, 2021). This study also met or exceeded the published standards cited in “The Guide for the Care and Use of Agricultural Animals in Research and Teaching” (ADSA-ASAS-PSA, 2020). The study protocol was established as appropriate for determination of utility and potential tissue concentrations and conducted pursuant to a food use authorization by the Food and Drug Administration Center for Veterinary Medicine.

Steers were housed and fed in 5.4 m × 15.8 m open-air, dirt-floored pens (5 steers/pen) without shade, equipped with automatic watering tanks for ad libitum freshwater access, and fed once daily for ad libitum intake as estimated using daily feed bunk inspections. The health status of each animal was monitored daily, and any unhealthy animals were identified and recorded via animal ID and treated according to an attending veterinarian’s recommendation.

### Experimental treatments

The five experimental cobalt treatments were formulated as a pelleted supplement containing one of the following: 1) cobalt carbonate (ICoNiChem Widnes Ltd, Windes, UK) fed to supply cobalt at 30 mg/steer/d, which meets or exceeds NASEM requirements in beef (2016) and dairy (2021); 2) cobalt acetate (Chemlock Metals, Cincinnati, OH) fed to supply cobalt at 30 mg/steer/d; 3) cobalt acetate fed to supply cobalt at 60 mg/steer/d; 4) cobalt lactate (Microbial Catalyst, Ralco Inc., Marshall, MN) fed to supply cobalt at 30 mg/steer/d; and 5) cobalt lactate fed to supply cobalt at 60 mg/steer/d (Table 1). Each supplement was incorporated into a base total mixed ration that was formulated to meet or exceed NASEM (2016) nutrient requirements for a 400 kg steer gaining 1.80 kg/d consuming 12.5 kg/d of dry matter (Table 2). Thus, the targeted added cobalt to the ration was 2.4 and 4.8 mg/kg, respectively, for the 30 and 60 mg/d. The experimental cobalt supplements were formulated by Ralco Nutrition, manufactured into 25 mm pellets, packaged in 23 kg bags by New Vision Cooperative (Worthington, MN), and shipped to the Agri Research Center, Canyon, TX. The experimental treatments were subsampled during manufacturing and sent to Midwest Laboratory (Omaha, NE) for cobalt testing (952.02) and retention samples were collected for future assays, if warranted.

**Table 1.** Ingredient composition of experimental treatment pellets containing three differing cobalt sources fed to growing steers at 30 or 60 mg of cobalt per steer per day

Ingredient^1^	Cobalt supplement composition and source fed at varying levels, mg/d				
	Carbonate	Acetate		Lactate	
	30	30	60	30	60
	(% of mix)				
Soybean meal	37.50	37.50	37.50	37.50	37.50
Limestone, 38% Ca	29.37	29.39	29.42	29.39	29.42
Urea, 46% N	13.93	13.93	13.93	13.93	13.93
Salt, white	10.15	10.15	10.15	10.15	10.15
Wheat midds	7.79	7.78	7.75	7.77	7.74
Unical limestone	0.36	0.35	0.32	0.35	0.31
Manganese sulfate, 32%	0.34	0.34	0.34	0.34	0.34
Zn oxide, 72%	0.20	0.20	0.20	0.20	0.20
Copper sulfate, 25.2%	0.14	0.14	0.14	0.14	0.14
ADE premix^2^	0.12	0.12	0.12	0.12	0.12
Selenium, 0.99%	0.07	0.07	0.07	0.07	0.07
EDDI, 79.5%	0.002	0.002	0.002	0.002	0.002
Cobalt carbonate, 50.5%	0.01	—	—	—	—
Cobalt acetate, 22%	—	0.03	0.06	—	—
Cobalt lactate, 22%	—	—	—	0.03	0.06

^1^Dry matter basis.

^2^Contains 110,230,000 IU/kg vitamin A, 11,023,000 IU/kg vitamin D, and 110,230 IU/kg of vitamin E.

**Table 2.** Ingredient and nutrient composition of total mixed rations fed to growing steers containing various cobalt sources at 30 or 60 mg cobalt per steer per day

Ingredients	Carbonate	Acetate		Lactate		SEM	*P* < ^1^
	30	30	60	30	60		
	(% of mix)						
Steam flaked corn	77.0	77.0	77.0	77.0	77.0	—	—
DDGS^2^	5.5	5.5	5.5	5.5	5.5	—	—
Alfalfa hay	8.5	8.5	8.5	8.5	8.5	—	—
Molasses Blend^3^	4.0	4.0	4.0	4.0	4.0	—	—
Micro premix^4^	1.0	1.0	1.0	1.0	1.0	—	—
Cobalt carbonate 30 pellet	4.0	—	—	—	—	—	—
Cobalt acetate 30 pellet	—	4.0	—	—	—	—	—
Cobalt acetate 60 pellet	—	—	4.0	—	—	—	—
Cobalt lactate 30 pellet	—	—	—	4.0	—	—	—
Cobalt lactate 60 pellet	—	—	—	—	4.0	—	—
*Nutrient composition*							
No. of samples	4	4	4	4	4	—	—
Dry matter, %	77.1	75.4	78.4	76.1	78.6	2.40	0.29
Crude protein, %	12.9	13.1	12.3	13.2	12.9	0.38	0.53
Ether extract, %	3.45	3.41	3.43	3.39	3.38	0.21	1.00
Ash, %	5.64	6.01	5.07	5.75	5.16	0.43	0.32
TDN^5^, %	84.4	84.0	85.0	84.2	84.8	0.56	0.28
NEg^6^, Mcal/kg	1.36	1.34	1.36	1.34	1.36	0.01	0.11
NEm^7^, Mcal/kg	2.03	2.01	2.04	2.03	2.04	0.02	0.28
Pellet cobalt, mg/kg	68.2^c^	67.9^cd^	127.3^a^	62.9^d^	119.0^b^	2.86	0.01
Feed cobalt, mg/kg	3.03^y^	3.66^y^	4.53^xy^	3.30^y^	5.38^x^	1.00	0.10

^1^Probably of significant F test for treatment.

^2^Dried distillers grains with solubles.

^3^Nutrabase 9, Westway Feed Products, LLC.

^4^Micro premix containing monensin and tylosan.

^5^Total digestible nutrients.

^6^Net energy gain.

^7^Net energy maintenance.

^a,b,c,d^Means within the same row with unlike superscripts differ, *P* < 0.05.

^x,y^Means within the same row with unlike superscripts differ, *P* < 0.10.

### Experimental design

One hundred Angus cross-bred steers weighing 385 ± 20 kg were used in a replicated 5 treatment double-blind randomized complete block design. Steers were blocked by initial body weight into a heavy body weight block and a light body weight block, then randomly allotted to treatment with two replications within each block. The steers were purchased from a single source originating from an eastern Oklahoma ranch, where steers had been backgrounded on a Coastal Bermudagrass pasture. The steers were transported 737 km from Bluejacket, OK, to the Agri-Research Center, Canyon, TX.

Steers arrived late afternoon on July 5, 2021, at the feed yard. The following morning steers were individually ear tagged, weighed, subcutaneously administered Bovi-Shield Gold 5 (Zoetis Inc., Parsippany, NJ), Vetrimec Plus (Bimeda-MTC Animal Health Inc., Cambridge, ON, Canada), and Vision 7 (Merck Animal Health USA, Rahway, NJ), and implanted with Revalor XS (Merck Animal Health USA, Rahway, NJ). After a 40-d acclimation period and following a 12-hr water shrink, steers were individually weighed on August 17 and reweighed on the following day after a second 12-h water shrink. Steers were stratified according to the August 17 body weight and allotted by body weight into a light or heavy block. Within each block, steers were randomly allotted to 10 pens with 5 steers/pen and the pens were randomly allotted to treatment, with each block having 2 pens per treatment (4 total pens per treatment). During the August 18 reweighing, each steer was ear tagged with a color-coded tag that corresponded to the color-coded treatment premix bags and the color-coded pens to which steers were designated. The mean body weight (August 17 and 18) was designated as initial body weight.

### Animal feeding

Steers were fed a finishing ration (Table 2) each morning for ad libitum consumption until cattle were harvested. The total mixed ration (TMR) including the appropriate cobalt supplement was mixed for 3 min using an Oswalt mixer (Roto-Mix LLC, Dodge City, KS) mounted on a truck. The mixer was flushed with 22.7 kg of hay between each experimental TMR mix to minimize cross contamination. Daily feed bunk scores were subjectively monitored prior to feeding each day and adjustments were implemented at each feeding to accomplish a clean feed bunk feeding strategy (Pritchard and Bruns, 2003). Orts, if present, were sampled daily from each pen and composited by weighing period and submitted for analysis of nutrient concentrations.

### Animal measurements and sampling

During the trial, cattle were weighed (Avery Weigh Tronix, Fairmont, MN; readability ± 0.45 kg, validated with certified weights before each use) without shrink in the morning prior to feeding every 28 d following the same pen sequence and at the time of harvest. One animal accidentally injured its stifle through inter-animal contact during weight collection in October which affected its mobility. Subsequent monitoring of this steer’s growth performance was deemed abnormal by the attending veterinarian and its data was removed from the data set. Average daily gain was calculated as (final body weight minus initial body weight)/d on feed.

### Animal harvest

Steers were harvested when each block was visually appraised to have approximately 12 mm of backfat thickness and the ability to grade low choice (USDA, 2017). The heavy block was harvested on November 18 after 92 d on feed, while the light block was harvested on December 13 after 117 d on feed. The day prior to harvest steers were individually weighed without shrink and 12 mL jugular blood was collected via 3 royal blue-top trace mineral vacutainer tubes (Becton-Dickinson, Franklin Lakes, NJ) containing no anti-coagulant. Blood samples were kept on ice until they were transported to the Texas A&M Veterinary Medical Diagnostic Laboratory (College Station, TX), and centrifuged at 3,300* × g* for 8 min at room temperature. Serum was decanted into clean labeled storage tubes and stored frozen at −20 °C until they were analyzed for serum cobalt and vitamin B_12_ concentrations. Due to laboratory error and disposal of the light block samples prior to analysis, only the heavy block samples were analyzed for serum cobalt.

At harvest time, each block was shipped via two trucks approximately 30 km to Tyson Fresh Meats, Inc. (Amarillo, TX) for harvest and subsequent carcass data collection. Hot carcass weights were recorded on harvest day. At harvest, 12 steers were randomly selected from each treatment regimen for collection of a minimum of 15 g of each of the five tissues (adipose, heart, kidney, liver, and muscle) immediately following hide removal using the procedures of Buttrey et al. (2013) and Jennings et al. (2020). The adipose tissue sample was collected from the ventral side of the epimysial connective tissue covering the pectoralis profundus. The heart sample was collected from the ventricular apex. The kidney sample was collected from approximately one-quarter of the left kidney containing the renal cortex. The liver sample was collected via slicing a 6 to 12 mm width ~12 mm inside the distal margin. The muscle sample was collected from the sternocephalicus in the neck. The tissue samples were partitioned into three equally sized subsamples for later analysis. All samples were kept on ice, transported to Texas A&M Veterinary Medical Diagnostic Laboratory, and stored frozen at −20 °C until they were analyzed.

### Carcass measurements

Carcasses were weighed on-line at the time of harvest (hot carcass weight) and then were evaluated by 3 trained individuals from the West Texas A&M University Beef Carcass Research Center for USDA Quality and Yield Grade (USDA, 2017). The ribeye area was determined by capturing a mirror image of the longissimus muscle with electrophoresis blotting paper and then measured electronically. Subcutaneous fat depth at the 12th rib was measured with a fat ruler and marbling score (10 = Practically Devoid, 30 = Slight, 50 = Modest, and 70 = Slightly Abundant) was visually assessed.

### Sample analyses

The experimental TMR for each pen was sampled at 28-d intervals and stored frozen. At the end of the study, the individual 28-d feed samples were thawed, hand-mixed, subsampled, and placed in a sample bag for shipping to the commercial testing laboratory. The 28-d orts samples were thawed, hand-mixed, subsampled and placed in a sample bag for shipping to the commercial testing laboratory. The 28-d samples were shipped to Midwest Laboratories (Omaha, NE) for proximate and mineral analyses using AOAC (2019) procedures. Monthly samples were analyzed for dry matter (935.29), crude protein (990.03), ether extract (Damon, 1966), ash (942.05), and cobalt (952.02). Energy calculations of total digestible nutrients, net energy gain, and net energy maintenance were according to NASEM (2016). Dry matter intakes were calculated as {[(feed delivered per pen *×* feed dry matter concentration) – (orts per pen *× *orts dry matter concentration)]/animals per pen}. Serum and tissue samples were analyzed via the trace mineral panel at Texas A&M Veterinary Medical Diagnostic Laboratory (TVDML; test 1070) for cobalt using inductively coupled plasma mass spectrometry following the procedures of Laur et al. (2020). Vitamin B_12_ was analyzed using an Immulite 2000 System Analyzer (Hicks et al., 1993; Siemens Corp., Washington, DC).

### Statistical analysis

All data were checked for normality and outliers using the UNIVARIATE procedure of SAS (version 9.4, SAS Institute Inc., Cary, NC) before any statistical analyses were conducted. Box-and-whisker plots and the Shapiro-Wilk test were used to verify that data were normally distributed (*P* > 0.15). All data were used for statistical analyses, i.e., identified outliers included. All data were then subjected to least-squares analysis of variance (ANOVA) for a randomized complete block design (Steele and Torrie, 1980) using the MIXED procedure of SAS with five treatments, two blocks, and two replicates per block. The following statistical model was used:


$$Y_{ij}=\text{ ​​}\mu \text{​​ }+{\text{Block}}_{i}+{\text{Trt}}_{j}+{\text{Block}}_{i}\times {\text{Trt}}_{j}+e_{ij}$$

where *Y*_*ij*_ is dependent variable, µ is overall mean, Block_i_ is light or heavy block based on body weight, Trt_j_ is treatment, and e_ij_ is residual random error.

Treatment, block, and treatment by block interaction were considered fixed effects with pen as the experimental unit. Individual animal carcass, serum, and tissue measurements were reduced to pen averages and statistically analyzed using pen as the experimental unit. Least squares means were separated by the PDIFF statement. The PDIFF statement uses the least significant difference method for separating treatment means when the ANOVA F-test was significant, *P* < 0.05 or indicating a tendency at *P* < 0.10. Three non-orthogonal contrast statements were used to evaluate responses to cobalt sources and inclusion rate: 1) inorganic cobalt vs. organic cobalt sources, 2) cobalt acetate vs. cobalt lactate, and 3) 30 vs. 60 mg/steer/d. Differences among treatment means were considered significant at *P* < 0.05 and tendencies at 0.05 ≤ *P* ≤ 0.10.

## Results and Discussion

### Feed composition

As expected, based on dietary formulation, the individual treatment nutrient concentrations were similar (*P* > 0.10) for dry matter, crude protein, ether extract, ash, and calculated net energy gain and net energy maintenance (Table 2). The experimental pelleted cobalt concentrations followed formulation expectations with significant (*P* < 0.05) differences in cobalt concentrations between the 30 and 60 mg/steer/d treatment supplement. Pelleted cobalt supplements at the 60 mg/steer/d treatment concentration were also significantly different (*P* < 0.05) from one another which was an unexpected result, however, can be explained via many factors. The variation in cobalt concentrations, in the experimental pellet and TMR can be related to several factors including ingredient variability, variability of feed sampling techniques, sorting during shipping or TMR mixing, and analysis error, etc. The targeted added cobalt was 2.4 and 4.8 mg/kg DM for the 30 and 60 mg/d, respectively. The TMR concentrations were slightly higher due to the combination of cobalt concentrations in the feed ingredients and the added targeted cobalt amounts. Total mixed ration cobalt concentrations for the cobalt carbonate, acetate and lactate at 30 mg/steer/d treatments tended to be similar (*P *< 0.10) as expected. Total mixed ration cobalt lactate at 60 mg/steer/d tended (*P* < 0.10) to be different than the 30 mg/steer/d cobalt sources with 60 mg/steer/d of cobalt acetate being intermediate. Although some variation is expected in conducting feed trace mineral analysis the resulting pellet and feed cobalt differences are still within statistical means based on formulation. Cobalt has been reported to be a difficult trace mineral to accurately measure in feeds due to sampling and analytical variation (Akins et al., 2013; Laur et al., 2020) even though dry ashing or wet ashing has shown excellent cobalt recoveries (Soon, 1998).

### Growth performance

The block effect in the statistical analysis was significant because the light block required an additional 25 d on feed to reach harvesting criteria. Contrasts of inorganic vs. organic cobalt sources, cobalt acetate vs. lactate, or inclusion rate (30 vs. 60 mg/steer/d) were similar (*P* > 0.10) for steers fed all treatments for starting, final, and body weight gains along with average daily gain, dry matter intake, and feed conversions (Table 3). No differences in individual treatments were detected (*P* > 0.10) among growth performance parameters indicating cobalt source or amount fed did not influence nor negatively affect growth performance, dry matter intake, and/or feed conversions. This study’s average daily gain was similar or greater compared with average daily gain previously reported in cobalt feeding studies (Buttrey et al., 2013; Araujo et al., 2019; Jennings et al., 2020), which resulted in these steers requiring shorter finishing times to reach market body weight than those documented in other published studies.

**Table 3.** Effects of cobalt concentration and source on performance of finishing steers

Item	Carbonate	Acetate		Lactate		SEM	Contrasts, *P* <		
	30	30	60	30	60		C vs O^1^	A vs L^2^	30 vs 60^3^
*n*, head	20	20	20	20	20				
Replicates	4	4	4	4	4				
Body weight, kg									
Start	458.3	458.1	445.7	461.1	453.9	23.5	0.96	0.80	0.62
Final	669.7	671.3	663.3	653.7	665.7	19.0	0.65	0.60	0.98
Gain	211.4	213.2	217.6	192.6	211.7	19.4	0.62	0.37	0.47
Daily gain, kg/d	2.03	2.05	2.09	1.85	2.04	0.17	0.52	0.27	0.33
Daily feed intake^4^, kg/d	11.0	10.8	11.0	10.7	10.8	0.32	0.49	0.60	0.79
Gain:feed^4^^,^^5^	0.18	0.19	0.19	0.17	0.19	0.01	0.64	0.20	0.19

^1^Carbonate (inorganic) vs organic (acetate and lactate).

^2^Cobalt acetate vs. cobalt lactate.

^3^Cobalt feeding level of 30 mg/steer/d vs. 60 mg/steer/d.

^4^On a dry matter basis.

^5^Gain:feed, kg body weight gain/kg feed intake.

Tiffany et al. (2003) and Tiffany et al. (2006) reported that feeding Angus steers required 0.14 mg/kg dry matter cobalt, although a finishing study (Tiffany and Spears, 2005) reported no benefits to increasing cobalt from 0.05 to 0.15 mg/kg dry matter on average daily gain and dry matter intake. In contrast, young rapidly growing cattle may be more sensitive to cobalt deficiency than older cattle. Schwarz et al. (2000) and Stangl et al. (2000) estimated that cobalt concentration should be 0.20 mg/kg dry matter in a corn silage diet for maximal average daily gain and dry matter intake. Dezfoulian and Aliarabi (2017) reported that feeding greater amounts of cobalt (0.25 vs. 0.5 mg/kg dry matter) increased feed intake in kid goats, but no differences were observed between cobalt sources, i.e., sulfate vs. glucoheptonate sources. Ammerman (1970) reported that carbonate, chloride, and sulfate cobalt forms were satisfactory dietary sources. Nagabhushana et al. (2005) reported that cobalt chloride up to 6 mg/kg dry matter was more efficacious in enhancing truly digestible organic matter than cobalt sulfate or acetate. Ammerman (1970) in a review of cobalt and copper for ruminants reported feeding cobalt to prevent a cobalt deficiency significantly increased body weight gain of finishing steers when added to a barley diet.

In contrast to nutrient requirements, cobalt supplementation has been implicated in enhancing fiber digestion in previous studies (Waterman et al., 2017; Casper et al., 2021; Jiao et al., 2021), but those studies were higher in forage concentrations compared to feedlot rations that are typically high grain and low forage rations. These research reports provide further indication that cobalt supplementation, both source and concentration, can have variable results on absorption and utilization in differing ruminant and production applications. Allen (1986) reported increased in vitro dietary cellulose digestibility when adding 10 mg/kg dry matter of cobalt from cobalt glucoheptonate. Lopez-Guisa and Satter (1992) supplemented 0.25 mg/kg dry matter of cobalt above NRC (NASEM 2016, 2021) recommendations and reported enhanced utilization of corn crop residue by growing heifers. Casper et al. (2021) reported lower ruminal ammonia and increased ruminal acetate concentrations when feeding 50 mg/cow/d of additional cobalt as cobalt lactate, which was associated with numerically greater total tract neutral detergent fiber digestion when lactating dairy cows were fed a 70% forage diet. Jiao et al. (2021) reported that feeding 4 or 7 g/d of cobalt lactate increased ruminal digestibility of dry matter, neutral detergent fiber, and hemicellulose. Scanning electron microscopy pictures visually demonstrated increased cellular and fiber digestion (Jiao et al., 2021).

The data reported herein indicate that growth performance and feed conversions for steers fed cobalt lactate were similar (*P* > 0.10) compared with steers fed cobalt carbonate and acetate, and all growth performance criteria were similar to industry expectations. The NRC (2005) specified 25 mg/kg dry matter cobalt as the maximum tolerable concentration, but no comments were reported regarding cobalt sources impacting the maximum tolerable concentration. The total cobalt intake (added plus basal) based on total mixed ration cobalt assay (Table 2) times DMI (Table 3) was 33.3, 39.5, 49.8, 35.3, and 58.1 mg/steer/d for 30 mg as cobalt carbonate, 30 mg as cobalt acetate, 60 mg as cobalt acetate, 30 mg as cobalt lactate, and 60 mg as cobalt lactate, respectively. Calculation using the pellet cobalt concentration, which represented added cobalt, times the inclusion rate (Table 2) times DMI resulted in an added cobalt intake of 30.0, 29.3, 56.0, 26.9, and 51.4 mg/steer/d, which meets or is close to formulation expectations for 30 mg as cobalt carbonate, 30 mg as cobalt acetate, 60 mg as cobalt acetate, but slightly below expectations for 30 mg as cobalt lactate and 60 mg as cobalt lactate, respectively. The discrepancies between formulation expectation and actual cobalt intake can be attributed to lower than expected DMI (12.5 vs. 11 kg/d; Table 3), mixing, sampling, and analytical variation. The total mixed ration fed in this study is well below the NRC maximum tolerable concentration of 25 mg/kg dry matter by approximately a factor of 10 and 6, respectively.

### Carcass measurements

The contrasts of inorganic vs. organic cobalt sources, cobalt acetate vs. lactate, or inclusion rates (30 vs. 60 mg/steer/d) were similar (*P* > 0.10) for steers fed all treatments for hot carcass weights, marbling scores, carcass yield grade, back fat thickness, and ribeye area (Table 4). Feeding cobalt lactate at either inclusion rate resulted in similar (*P* > 0.10) carcass measurements when compared with other cobalt sources and/or inclusion rates. Thus, cobalt lactate demonstrated no changes in carcass measurements and would be an acceptable alternative cobalt source to carbonate and acetate for feeding beef and dairy cattle.

**Table 4.** Effects of cobalt concentration and source on carcass characteristics of finished steers

Item	Carbonate	Acetate		Lactate		SEM	Contrasts, *P* <		
	30	30	60	30	60		C vs O^1^	A vs L^2^	30 vs 60^3^
*n*, head	20	20	20	20	20				
Replicates	4	4	4	4	4				
Hot carcass, kg	404.9	406.2	405.6	398.3	408.0	11.25	0.81	0.75	0.64
Marbling score^4^	50.1	46.3	47.0	48.5	49.5	2.06	0.23	0.20	0.98
USDA yield grade^5^	3.41	3.27	3.37	3.36	3.29	0.18	0.60	0.99	0.87
Back fat, cm	1.43	1.29	1.39	1.38	1.31	0.18	0.60	0.99	0.87
Ribeye area, cm^2^	101.8	102.1	101.7	98.5	100.1	3.21	0.50	0.33	0.97

^1^Carbonate (inorganic) vs organic (acetate and lactate).

^2^Cobalt acetate vs. cobalt lactate.

^3^Cobalt feeding level of 30 mg/d vs. 60 mg/d.

^4^Marbling score: 10 = practically devoid, 30 = slight, 50 = modest, and 70 = slightly abundant.

^5^ USDA (2017).

### Serum cobalt and vitamin B_12_

Cobalt is an essential component of vitamin B_12_ and is surrounded by six linked groups to form an octahedron (NRC, 2005). In the ruminant, vitamin B_12_ can be synthesized from inorganic cobalt by the ruminal bacteria and is a required nutrient for propionate and folate metabolism in both ruminant and non-ruminant species. As a result, cobalt detected in the serum and tissues is predominately in the form of vitamin B_12_.

The contrast of inorganic vs. organic cobalt sources and cobalt acetate vs. lactate were similar (*P* > 0.10) for serum cobalt concentrations, whereas the contrast for feeding greater cobalt intakes increased (*P* < 0.02) serum cobalt concentrations by 100% for steers fed 60 mg/steer/d compared with steers fed 30 mg/steer/d (Table 5). The separation of means indicated that steers fed 60 mg/steer/d of cobalt had greater (*P *< 0.05) serum cobalt concentrations compared with steers fed 30 mg/steer/d cobalt with no differences detected among cobalt sources. Tiffany et. al., (2006) recorded that plasma vitamin B_12_ concentrations were greatly increased when cobalt concentration was increased from 0.10 to 1.0 mg/kg of DM in both growing and finishing cattle. Cobalt source, either from cobalt carbonate or cobalt propionate, did not affect plasma B_12_ concentration, only concentration fed. In agreement, Waterman, et. al., (2017) in which beef cattle supplemented with cobalt carbonate or cobalt glucoheptonate reported no effect on serum cobalt concentrations when fed to achieve the same amount of cobalt supplementation. Serum cobalt concentrations found in this study (Table 5) fall within the normal serum cobalt range according to Puls (1994). It should be noted that this range of 0.9 to 15 ng/mL includes all treatment serum cobalt concentrations found in the current study as acceptable.

**Table 5.** Effects of cobalt concentration and source on serum cobalt and tissue concentration of finishing steers

Item	Carbonate	Acetate		Lactate		SEM	Contrasts, *P* <		
	30	30	60	30	60		C vs O^1^	A vs L^2^	30 vs 60^3^
*n*, head	20	20	20	20	20				
Replicates	4	4	4	4	4				
*Serum*									
Serum cobalt^4^, ng/mL	6.66^b^	6.77^ab^	13.83^a^	6.32^b^	12.17^a^	2.79	0.96	0.64	0.02
Serum B_12_, pg/mL	179.4	211.8	209.5	207.7	192.3	37.9	0.26	0.64	0.95
*Tissue, dry matter basis*									
Liver, ng/g	450.8^c^	718.0^bc^	937.7^b^	502.2^c^	1535.7^a^	230.2	0.08	0.24	0.01
Kidney, ng/g	282.6^c^	346.6^c^	821.2^b^	360.3^c^	934.8^a^	83.6	0.22	0.25	0.01
Heart, ng/g	107.5	182.2	190.6	99.1	196.5	91.3	0.53	0.53	0.22
Muscle, ng/g	45.2^b^	76.6^ab^	69.7^ab^	51.8^b^	107.8^a^	18.3	0.10	0.69	0.06
Adipose, ng/g	4.27^c^	5.43^bc^	8.94^ab^	6.21^c^	9.77^a^	1.74	0.22	0.50	0.01

^1^Carbonate (inorganic) vs organic (acetate and lactate).

^2^Cobalt acetate vs. cobalt lactate.

^3^Cobalt feeding level of 30 mg/d vs. 60 mg/d.

^4^Serum cobalt analyzed only for the heavy block of steers, *n* = 2.

^a,b,c^Means within the same row with unlike superscripts differ, *P* < 0.05.

There are differences noted between ruminants and their production type, as well as ration fed in relation to serum cobalt concentrations. Research shows that serum cobalt concentrations can vary when concentrations are measured in dairy cattle across all production stages—gestating, lactating, or neither (Kincaid et al., 2003; Kincaid and Socha, 2007; Akins et al., 2013). The serum, or plasma, cobalt concentrations in these studies varied in response to animal age, stage of production, supplementation amount, and length of supplementation. Additionally, previous research has shown that serum cobalt concentrations in dairy cattle were sensitive to cobalt source and insensitive to supplementation amount both of which contrast with the current study. Akins et al. (2013) reported greater plasma cobalt concentrations when lactating dairy cattle were fed an organic cobalt source (glucoheptonate) compared with cobalt carbonate indicating that organic cobalt forms are more bioavailable than inorganic forms. amounts of cobalt supplementation in dairy cattle, up to 75 mg/cow/d, resulted in serum cobalt concentrations within each study that were similar across supplementation amounts indicating that serum cobalt concentrations were influenced by study characteristics other than amount of cobalt supplementated (Kincaid et al., 2003; Kincaid and Socha, 2007; Akins et al., 2013).

The contrasts of inorganic vs. organic cobalt sources, cobalt acetate vs. cobalt lactate, and cobalt inclusion rate (30 vs. 60 mg/steer/d) were similar (*P* > 0.10) for serum vitamin B_12_ concentrations (Table 5). These data and that of Akins et al. (2013) indicate that inorganic and organic cobalt forms are similar in serum vitamin B_12_ response when fed at typical inclusion rates to meet the animal’s cobalt requirement to achieve adequate vitamin B_12_ status. In addition, several dairy cattle studies support the lack of response of serum or plasma vitamin B_12_ concentrations in response to cobalt supplementation (Kawashima et al., 1997a; Kincaid et al., 2003; Kincaid and Socha, 2007). Akins et al. (2013) reported a tendency for reduced plasma vitamin B_12_ concentrations in response to higher amounts of cobalt glucoheptonate. In contrast, Dezfoulian and Aliarabi (2017) also reported that feeding increasing cobalt amounts resulted in increased ruminal propionate concentrations and vitamin B_12_ synthesis, which increased serum vitamin B_12_ concentrations that led to increased serum glucose concentrations via methylmalonyl CoA mutase. Research has reported that ruminal synthesis of vitamin B_12_ is increased when feeding higher dietary cobalt concentrations (Mills, 1981; Tiffany et al., 2006). Tiffany and Spears (2005) demonstrated a grain source effect on ruminal vitamin B_12_ synthesis showing that steers fed corn required greater cobalt concentrations vs. steers fed barley. In contrast, research done in cattle given higher forage rations and fed additional cobalt resulted in greater ruminal acetate concentrations with no change in propionate concentrations (Casper et al., 2021; Jiao et al., 2021).

Cobalt supplementation may have different impacts in the ruminant animal depending on whether ruminal or post-ruminal metabolism is evaluated. Tiffany et al. (2003) showed a linear effect on liver B_12_ concentrations as inclusion rate increased between cobalt carbonate and cobalt propionate in finishing steers. A significant difference in liver B_12_ concentration was found between no cobalt supplementation and increasing amounts of cobalt carbonate as well as increasing amounts of cobalt propionate in finishing steers. Tiffany and Spears (2005) reported no benefits to increasing cobalt in the form of cobalt carbonate from 0.05 to 0.15 mg/kg dry matter on liver B_12_ concentrations. However, there was a significant difference between no added cobalt and cobalt addition of 0.05 and 0.15 mg/kg DM. Stangl et al. (2000) reported that increasing dietary cobalt concentrations resulted in increasing liver concentrations of vitamin B_12_. Schwarz et al. (2000) and Stangl et al. (2000) estimated the cobalt concentration to be 0.20 mg/kg dry matter on a corn silage diet for maximal plasma and liver B_12_ concentrations. The plasma vitamin B_12_ concentrations in the present study are similar to vitamin B_12_ concentrations published by Stangl et al. (2000), Kincaid and Socha (2007), Akins et al. (2013), and González-Montaña et al. (2020). Stangl et al. (2000) further stated that the cobalt dietary concentration required to minimize homocysteine and methylmalonic acid in plasma and to maximize vitamin B_12_ and folate status was greater than earlier NRC (1996) recommendations, which is why NASEM (2016) increased dietary cobalt recommendations. The serum B_12_ concentrations in this study (Table 5) indicate that plasma B_12_ concentrations in steers are slightly lower than values reported by Akins et al. (2013) and Kincaid and Socha (2007). These values are also lower than Puls (1994) would indicate as cobalt being adequate for serum B_12_ concentrations in cattle. Cobalt acetate at both 30 and 60 mg/steer/d and cobalt lactate at 30 mg/steer/d fell within marginal cobalt status according to Puls (1994) with the remaining concentrations falling just short of being within the cobalt marginal range (0.25 to 0.35 ng/mL). These values are also much lower than those reported by Kincaid et al. (2003) from lactating dairy cows as would be expected given that the metabolism of a lactating dairy cow is vastly different compared with a growing steer. Elliot et al. (1979) established that serum vitamin B_12_ concentrations are reduced during early lactation and Kincaid et al. (2003) confirmed the early lactation decrease and reported further reductions of serum B_12_ concentrations at day 120 in milk. Liu et al. (2021) reported that increasing cobalt supplementation to calves increased serum vitamin B_12_ concentrations.

### Tissue concentrations

Cobalt is distributed throughout the body, primarily as vitamin B_12_-bound cobalt (NRC, 2005). The liver and kidney are the edible tissues with the highest concentration of cobalt although the musculature is the largest total storage tissue with about 43% of the total body cobalt concentration (NRC, 2005; EFSA, 2012). The liver is also the storage site of vitamin B_12_. In addition, the liver and kidney are the organs that best reflect dietary cobalt intake (Henry et al., 1997; NRC, 2005; EFSA, 2012). However, cobalt retention in tissues is very low in ruminants. Looney et al. (1976) estimated that 71% to 85% of orally supplemented ^60^Co was excreted by sheep within 48 hours, with virtually all of the dose excreted within 5 d. Liver cobalt and liver vitamin B_12_ concentrations have been shown to be an accurate measurement of dietary cobalt (Stangl et al., 2000). Both liver and serum cobalt concentrations are tested concurrently as there can be variability in the proportion of vitamin B_12_ found in the serum and liver tissue that is not associated with just the cobalt concentration making interpretations of just one cobalt source of testing difficult (Herdt and Hoff, 2011).

The contrast of inorganic vs. organic cobalt sources indicated that the organic forms demonstrated a tendency (*P* < 0.08) for greater liver cobalt concentrations, while the cobalt acetate vs. cobalt lactate contrast was not significant (*P* > 0.10), demonstrating that both organic sources are similar in bioavailability (Table 5). The contrast of cobalt inclusion rate (30 vs. 60 mg/steer/d) indicated that feeding more cobalt resulted in greater (*P* < 0.01) liver cobalt concentrations with the 60 mg/steer/d treatments resulting in over twice as much liver cobalt as the 30 mg/steer/d treatments. The liver cobalt concentrations were greater (*P* < 0.05) for steers fed 60 mg/steer/d cobalt as lactate compared with steers fed 60 mg/d cobalt as acetate which were greater (*P* < 0.05) than steers fed 30 mg/d cobalt as acetate which were greater (*P* < 0.05) than steers fed 30 mg/d cobalt as carbonate or lactate (Table 5). The liver cobalt concentrations are of special interest because the liver is an edible tissue and the primary storage location with greater cobalt concentrations than other tissues (Jorhem et al., 1989; EFSA, 2012). In addition, younger animals tended to have lower liver cobalt concentrations than older animals (Counotte et al., 2019). Data from the current study indicated that liver concentrations are much higher than concentrations reported for cattle with little or no trace mineral supplementation (Jorhem et al., 1989; López Alonso et al., 2004; Counotte et al., 2019). Liver cobalt concentrations in the current study are within the ranges (200 to 21, 600 ng/g) reported with cattle, sheep, or lamb cobalt supplementation (Puls, 1994; Henry et al., 1997; Kawashima et al., 1997a; Kincaid et al., 2003; Kincaid and Socha, 2007; Akins et al., 2013). The greater liver cobalt concentrations in the current study for organic cobalt sources may indicate the greater cobalt bioavailability when feeding organic sources compared with inorganic sources, which is supported by the results of Dezfoulian and Aliaribi (2017). Thus, liver cobalt concentrations will be a function of cobalt intake and cobalt source solubility. These data also demonstrate that liver cobalt concentrations are lower for finishing steers compared to lactating dairy cows, which ranged from 1,100 to 2,500 ng/g (Kincaid et al., 2003; Kincaid and Socha, 2007; Akins et al., 2013). In addition, the doubling of liver cobalt concentrations with greater supplementation is similar in magnitude to results observed for sheep (Henry et al., 1997; Kawashima et al., 1997a) but not in lactating dairy cows. In lactating dairy cows, increasing cobalt supplementation amounts above requirements did not have the dramatic impact on liver cobalt concentrations observed with beef cattle (Kincaid et al., 2003; Kincaid and Socha, 2007; Akins et al., 2013). The lack of response for liver cobalt concentrations in lactating dairy cattle may be the result of deposition of vitamin B_12_ in milk and the major impacts of parity and length of lactation on cobalt metabolism.

The inorganic vs. organic cobalt sources contrast indicated that kidney cobalt concentrations were similar (*P *> 0.10) when organic sources were compared with carbonate with both organic sources demonstrating similar (*P* > 0.10) kidney cobalt concentrations (cobalt acetate vs. cobalt lactate; Table 5). The contrast of cobalt inclusion rate (30 vs. 60 mg/steer/d) demonstrated (*P* < 0.01) that feeding greater amounts of cobalt resulted in a doubling of kidney cobalt concentrations similar to the changes observed in the liver. The kidney cobalt concentrations were greater (*P* < 0.05) for steers fed 60 mg/steer/d cobalt as acetate or lactate compared with steers fed 30 mg/steer/d cobalt as cobalt acetate. Steers fed 30 mg/steer/d cobalt as carbonate had the lowest kidney cobalt concentrations with steers fed 30 mg/steer/d cobalt as lactate being intermediate and similar (*P* > 0.10) between 30 mg/steer/d cobalt as carbonate and acetate. Urinary excretion by the kidney is the main route of cobalt excretion with small amounts of fecal endogenous losses (NRC, 2001, 2005). Jorhem et al. (1989) reported that kidney cobalt concentrations collected from ruminant animals at a slaughterhouse, along with compiled literature data, would be four to five times lower than liver cobalt concentration and approximately four to five times greater than meat/muscle cobalt concentrations. However, more recent slaughterhouse studies with more animals indicated that kidney cobalt concentrations were about half of liver cobalt concentrations (López Alonso et al., 2004). In addition, studies evaluating cobalt supplementation in sheep confirmed that liver and kidney cobalt concentrations are much greater with cobalt supplementation than those measured in slaughter surveys where supplementation status was unknown, and that liver cobalt concentrations were about twice the concentration found in kidneys (Henry et al., 1997; Kawashima et al., 1997a). The kidney cobalt concentrations in the current study were similar to those reported by Henry et al. (1997) and Kawashima et al., (1997a) for sheep wethers which ranged from 220 to 6510 ng/g cobalt.

The contrasts of inorganic vs. organic cobalt sources, cobalt acetate vs. cobalt lactate, and cobalt inclusion rate (30 vs. 60 mg/steer/d) were similar (*P* > 0.10) for heart tissue cobalt concentrations indicating that carbonate, acetate, and lactate forms of cobalt had similar influences on heart cobalt concentrations (Table 5) and feeding more cobalt does not increase heart muscle cobalt concentrations. These results are within the range of heart muscle cobalt concentrations (100 to 2,500 ng/g) reported by Henry et al. (1997) and Kawashima et al., (1997a) who reported that greater cobalt inclusion rates resulted in greater heart cobalt concentrations. Henry et al. (1997) also reported that increasing days on feed beyond 40 d within cobalt feeding rate resulted in similar heart cobalt concentrations. Henry et al. (1997) and Kawashima et al. (1997a) demonstrated that heart cobalt concentrations can be ~5 times greater than skeletal muscle cobalt concentrations.

The inorganic vs. organic cobalt source contrast for muscle indicated only a tendency (*P* < 0.10) for organic cobalt sources to increase muscle cobalt concentrations compared with cobalt carbonate, while the cobalt acetate vs cobalt lactate contrast indicated that muscle cobalt responses were similar (*P* > 0.10) between the organic cobalt sources. The contrast of cobalt inclusion rate (30 vs. 60 mg/steer/d) indicated that feeding more cobalt resulted in 51% greater muscle cobalt concentrations. Muscle cobalt concentrations were greater for steers fed 60 mg/steer/d cobalt as lactate compared with steers fed 30 mg/steer/d cobalt as carbonate or lactate with steers being fed the remaining treatments being similar (*P* > 0.10) and intermediate. These muscle cobalt concentrations are in alignment with those reported by Henry et al. (1997) and Kawashima et al., (1997a) which ranged from 49 to 260 ng/g. Approximately 43% of the body cobalt is stored in muscle (EFSA, 2012). Although Jorhem et al. (1989) reported that meat (muscle) cobalt concentrations are very low at ≤ 0.003 mg/kg fresh weight, more recent slaughterhouse studies indicated that muscle cobalt concentrations were up to 73 ng/g (López Alonso et al., 2004). The association of muscle cobalt concentrations as vitamin B_12_ with the methylmalonyl-CoA mutase enzyme suggests greater support for the conversion of propionate to glucose to enhance muscle tissue deposition (Majdoub et al., 2003). The speculation that muscle glycogen concentrations could be increased was not measured in the current study. Bishop et al. (1991) reported that feeding 7 g/d of cobalt dextrose lactone in gestating nonlactating beef cows increased plasma glucose concentrations. Liu et al. (2021) reported that increased cobalt supplementation increased blood glucose concentrations.

The contrast of inorganic compared with organic cobalt sources indicated that adipose tissue cobalt concentrations were similar (*P* > 0.10) for organic sources compared with inorganic cobalt carbonate, whereas the contrast of cobalt acetate vs lactate was similar (*P* > 0.10), indicating similar response with both organic cobalt sources (Table 5). The contrast of cobalt inclusion rate (30 vs. 60 mg/steer/d) indicated that feeding more cobalt can increase (*P* < 0.01) adipose tissue cobalt concentrations. Adipose tissue cobalt concentrations were greater (*P* < 0.05) for steers fed 60 mg/steer/d cobalt from cobalt lactate and both inclusion amounts of cobalt acetate compared with steers fed 30 mg/steer/d cobalt as cobalt carbonate or lactate with the remaining treatment being intermediate (Table 5). To our knowledge no prior literature data exists on adipose tissue cobalt concentrations.

In summary, serum cobalt concentrations were within normal range as referenced by Puls (1994) for all cobalt sources and concentrations supplemented. Vitamin B_12_ concentrations observed in this study were lower than previously reported while tissue cobalt concentrations were within the ranges previously reported for cattle and sheep supplemented with a variety of cobalt forms (Henry et al., 1997; Kawashima et al., 1997a; Kincaid et al., 2003; Kincaid and Socha, 2007; Akins et al., 2013).

## Conclusions

This study demonstrated that cobalt source, whether inorganic or organic, and cobalt inclusion rate, whether 30 or 60 mg/steer/d, resulted in similar feedlot steer growth performance and carcass measurements. However, organic cobalt sources increased liver, kidney, and muscle cobalt concentrations with responses being similar for both acetate and lactate. Feeding more cobalt (60 mg/steer/d vs. 30 mg/steer/d) increased liver, kidney, muscle, adipose, and serum cobalt concentrations; however, these tissue concentrations were within physiological concentrations previously observed with supplementation studies. Supplementing cattle feed with cobalt lactate, which is not currently approved for use in the United States, resulted in similar growth performance, carcass characteristics, and tissue cobalt concentrations compared with cobalt carbonate and cobalt acetate, which are cobalt sources approved for inclusion in cattle feeds. As a result, cobalt lactate can be fed without any adverse effect and meets the ruminant’s cobalt nutrient requirement as shown through growth performance, carcass characteristics, and tissue cobalt concentrations.
